# Investigating SARS-CoV-2 Susceptibility in Animal Species: A Scoping Review

**DOI:** 10.1177/11786302221107786

**Published:** 2022-06-28

**Authors:** Connor Rutherford, Pratap Kafle, Catherine Soos, Tasha Epp, Lori Bradford, Emily Jenkins

**Affiliations:** 1 School of Public Health, University of Saskatchewan, Saskatoon, SK, Canada; 2 Department of Veterinary Microbiology, Western College of Veterinary Medicine, University of Saskatchewan, Saskatoon, SK, Canada; 3 Department of Veterinary Biomedical Sciences, Long Island University Post Campus, Brookville, NY, USA; 4 Ecotoxicology and Wildlife Health Division, Science & Technology Branch, Environment and Climate Change Canada, Saskatoon, SK, Canada; 5 Department of Veterinary Pathology, Western College of Veterinary Medicine, University of Saskatchewan, Saskatoon, SK, Canada; 6 Department of Large Animal Clinical Sciences, Western College of Veterinary Medicine, University of Saskatchewan, Saskatoon, SK, Canada; 7 Ron and Jane Graham School of Professional Development, College of Engineering, and School of Environment and Sustainability, University of Saskatchewan, Saskatoon, SK, Canada

**Keywords:** Epidemiological methods, in silico, in vitro, in vivo, scoping review, SARS-CoV-2

## Abstract

In the early stages of response to the SARS-CoV-2 pandemic, it was imperative for researchers to rapidly determine what animal species may be susceptible to the virus, under low knowledge and high uncertainty conditions. In this scoping review, the animal species being evaluated for SARS-CoV-2 susceptibility, the methods used to evaluate susceptibility, and comparing the evaluations between different studies were conducted. Using the PRISMA-ScR methodology, publications and reports from peer-reviewed and gray literature sources were collected from databases, Google Scholar, the World Organization for Animal Health (OIE), snowballing, and recommendations from experts. Inclusion and relevance criteria were applied, and information was subsequently extracted, categorized, summarized, and analyzed. Ninety seven sources (publications and reports) were identified which investigated 649 animal species from eight different classes: Mammalia, Aves, Actinopterygii, Reptilia, Amphibia, Insecta, Chondrichthyes, and Coelacanthimorpha. Sources used four different methods to evaluate susceptibility, *in silico, in vitro, in vivo*, and epidemiological analysis. Along with the different methods, how each source described “susceptibility” and evaluated the susceptibility of different animal species to SARS-CoV-2 varied, with conflicting susceptibility evaluations evident between different sources. Early in the pandemic, *in silico* methods were used the most to predict animal species susceptibility to SARS-CoV-2 and helped guide more costly and intensive studies using *in vivo* or epidemiological analyses. However, the limitations of all methods must be recognized, and evaluations made by *in silico* and *in vitro* should be re-evaluated when more information becomes available, such as demonstrated susceptibility through *in vivo* and epidemiological analysis.

## Introduction

The severe acute respiratory syndrome coronavirus 2 (SARS-CoV-2) needs no introduction; it has caused the deadliest pandemic in recent human history. The virus was first detected in December 2019, from ill individuals in Wuhan, located in Hubei, a province in China. In January, scientists determined that the causative agent was a novel coronavirus (CoV). Due to its high sequence similarity with Severe Acute Respiratory Syndrome coronavirus (SARS-CoV-1), the International Committee on Taxonomy of Viruses classified this new virus as SARS-CoV-2 and the disease was named COVID-19.^1,2^ SARS-CoV-2 rapidly spread throughout the world, labeled as a public health emergency of international concern on January 30th and then a pandemic on March 11th by the World Health Organization.^3^ As of May 20, 2022, worldwide, there have been 521,920,560 and 6,274,323 confirmed cases and deaths from COVID-19.^4^

CoVs are a positive sense, non-segmented RNA virus from the Coronaviridae family and Coronavirinae subfamily.^5-7^ Within the Coronavirinae subfamily, there are four genera of CoVs: alpha, beta, delta, and gamma.^8,9^ SARS-CoV-2 belongs to the betacoronavirus genera.^8,9^ Alpha and betacoronaviruses mainly infect mammals while delta and gammacoronaviruses infect mostly birds, with the exception of a pig and beluga whale CoV which are found in the delta and gamma genera, respectively.^8-10^

Besides SARS-CoV-2, there are six additional CoVs that cause disease in humans, HCoV-NL63, HCoV-OC43, HCoV-229E, HKU1, Middle Eastern Respiratory Syndrome (MERS), and SARS-CoV-1, all of which are zoonotic in origin.^11-14^ The most pathogenic CoVs to humans are SARS and MERS; both of these CoVs originated from bats and their “intermediate hosts,” or more appropriately, bridging hosts which spread the virus to people, are the civet cat and dromedary camel.^15-17^ Although the origin of SARS-CoV-2 is still being debated, it has been hypothesized that SARS-CoV-2 is the result of a homologous recombination event occurring between a bat and pangolin CoV.^18^ Novel CoVs also continue to be discovered; for example, CCoV-HuPn-2018 isolated from a child with pneumonia in Sarawak Malaysia.^19^

The host tropism for SARS-CoV-2 is dependent on its spike (S) protein, which binds to and facilitates entry into host cells. The S1 domain of the spike protein binds to the host receptor Angiotensin-Converting Enzyme 2 (ACE2) through its receptor binding domain (RBD), after which the S2 domain facilitates viral fusion and entry, which is primed by the protease TMPRSS2.^17,20-22^ The human ACE2 (hACE2) receptor is a type I membrane protein and is normally involved in the renin angiotensin system, cleaving angiotensin I into angiotensin 1-9 and angiotensin II into angiotensin 1-7.^23,24^ The ACE2 receptor is also utilized by SARS-CoV-1; however, SARS-CoV-2 binds the ACE2 receptor with a higher affinity, leading to higher rates of infection and transmission.^25,26^

As SARS-CoV-2 is a zoonotic novel pathogen, early in the pandemic it was a priority to determine which animal species may be susceptible to the virus. Animals that are susceptible to SARS-CoV-2 can serve as models in therapeutics or vaccine trials, and targets for further investigation for epidemiological and ecological studies to determine which animal(s) serve as intermediate (bridging) or reservoir hosts, potentially allowing for the continued spread and occurrence of mutations. Spillover and spillback of SARS-CoV-2 has already occurred on a mink farm in the Netherlands.^27,28^

An animal species’ susceptibility to SARS-CoV-2 can be established through four different methods: *in silico, in vitro, in vivo*, and epidemiological analysis.^29^ In general, *in silico* analysis refers to using computer modeling or simulations to evaluate receptor binding; *in vitro* analysis refers to investigating receptor binding or viral entry in cell lines; *in vivo* analysis refers to testing for antibodies and/or RNA of the virus in experimentally exposed live animals; and epidemiological analysis refers to testing for the presence of antibodies and/or RNA of the virus in naturally infected animals.^29-38^

Studies evaluating animal susceptibility to SARS-CoV-2 are emerging at a rapid rate. Due to the influx of literature evaluating the susceptibility of animal species to SARS-CoV-2, a scoping review was conducted to determine which animal species were being investigated, the methods used to evaluate susceptibility, and the conclusions regarding the susceptibility of different classes and species of animals, in order to help identify targets for ongoing surveillance and epidemiological studies. Also, how different susceptibility predictions can vary between sources is expressed. We also suggest criteria which can be applied for weighing evidence of animal susceptibility to an emerging zoonoses, even for a novel pathogen under high scientific uncertainty.

## Methods

The framework for the scoping review was based on the Preferred Reporting Items for Systematic Reviews and Meta-Analysis Extension for Scoping Reviews (PRISMA-ScR).^39^

### Search strategy

Sources (publications or reports) were collected between July 9th-13th, 2020 and December 30th-January 2nd, 2021, from established databases (Medline, Scopus, Web of Science, PubMed, Global Health, and Public Health Database), and the first 100 results from Google Scholar collected on a single day in both time frames. For the databases and Google Scholar, search terms were drafted and then reviewed by a university librarian and an interdisciplinary research team (epidemiologist, microbiologist, and social scientist) for input and modification. Additional sources were added through investigating cited references in the selected sources (snowballing), from the recommendations of expert researchers, and the World Organization for Animal Health (OIE).^40^ For OIE, sources were gathered on April 30, 2021 and were found by accessing the *COVID-19 Events in Animals* webpage.^40^ All sources were imported into Zotero software and duplicates were removed manually.^41^ An example of the search strategy is shown in Supplemental Figure S1.

### Eligibility criteria

Eligible sources consisted of peer-reviewed or gray literature (pre-prints or non-peer reviewed articles) that investigated or reported on an animal species’ susceptibility to SARS-CoV-2. Articles that were excluded include, self-described review articles, studies using animal models to evaluate SARS-CoV-2 therapeutics or vaccines, studies using lab specific or transgenic animals, articles not in English, or duplicate studies reporting on the same naturally infected animals in time and space such as the SARS-CoV-2 outbreaks on the mink fur farms, in which case the formal report to the OIE took precedence.

### Selection of sources

**Figure 1.**
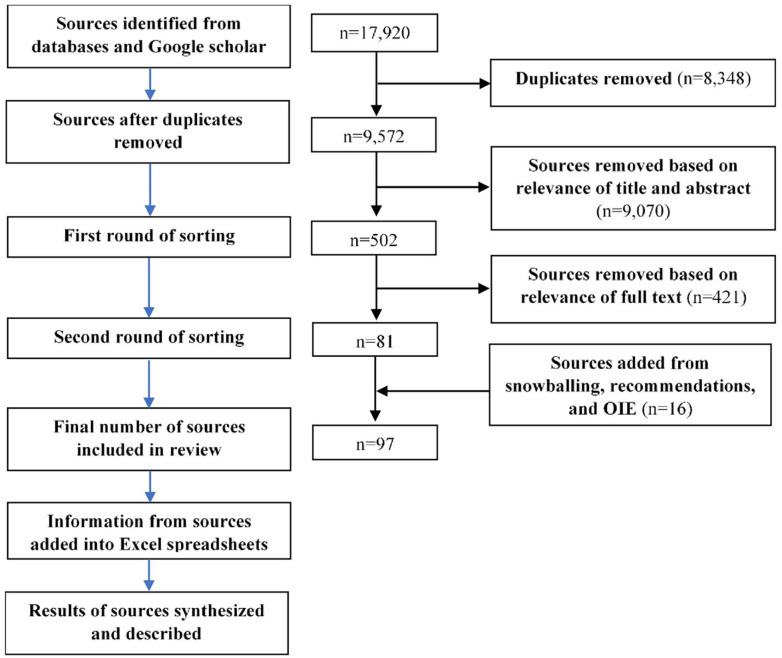
Flow chart demonstrating the methods used for source gathering, selection, and synthesis for the scoping review.

After duplicates were removed, sources were sorted by two researchers in two rounds, in which irrelevant sources were removed (Figure 1). The first round consisted of reading the title and abstract of each source. If no abstract was provided, the title and keywords were used. The next round comprised of reading the source material. After both rounds, the researchers then compared their results, and any disagreements (n = 555) were settled through consensus. In a scoping review, settling disagreements through consensus has shown to be an effective method as described by Peterson et al.^42^ After the second round, the sources selected underwent snowballing. Sources based on recommendations from researchers (often seminal or novel findings) were added throughout the scoping review process, and subsequently underwent snowballing. Additionally, after the second round, results from animals naturally infected with SARS-CoV-2 were compiled from OIE.

### Data charting

Once the selected sources were finalized, corresponding information from each source was entered into predetermined categories in two Excel spreadsheets. The first Excel spreadsheet categories were: author, title of source, date published/uploaded, source type (self-described by source, including dispatches, letters, articles, reports, etc.), country of first author, method used to evaluate susceptibility, overview of the methods, number of animal species evaluated, and overview of findings. The second spreadsheet contained a list of all animal species investigated with the animal’s taxonomic class, scientific and common name, which were matched with the investigating source.

The scientific and common name were identified through an accession number or sequence ID provided from the source linking to a public database such as the National Centre for Biotechnology Information.^43^ The taxonomic class, if not already provided by the source, was found through the Integrated Taxonomic Information System.^44^ If no sequence ID was provided, the scientific name and common name in the source were used. If the common name and scientific name did not match, the common name took priority that is *in vivo* studies citing *Canis lupus* were presumed to be using dogs versus wolves. If only the common name was provided it was matched to its representative scientific name, where possible. This was dependent upon the common name being linked to a single species, such as cats or dogs (*Felis catus* and *Canis lupus domesticus*). If the common name was too general and could not be matched to a specific species, then all animal species which shared the similar common name were identified in the Excel spreadsheet and the unstated species was assumed to be the species most commonly investigated by the other sources. For example, if the common name listed was “bear,” and there were four studies on American black bears, 11 on brown bears, and 12 on polar bears, a source using only the common name “bear” was entered as polar bear (*Ursus maritimus*). As the location where the source study occurred was not considered, this is an acknowledged limitation of the scoping review. Subspecies were removed, recording only the genus and species. For example, if a source investigated related subspecies such as *Sus scrofa* and *Sus scrofa domesticus*, only *Sus scrofa* would have been recorded and that source would be considered to have investigated only one species. Only certain subspecies were included, namely *Canis lupus familiaris* (dog) and *Canis lupus dingo* (dingo), and *Mustela putorius furo* (Ferret) and *Mustela lutreola biedermanni* (Mink) as there were a large number of sources that investigated these animals and made clear distinctions among subspecies. Humans were not included in the animal species list and were not counted.

### Synthesis of results

Descriptive statistics summarizing source characteristics, animal species, and their corresponding class, the methods used for evaluating an animal’s susceptibility, the conclusion of the source regarding susceptibility of certain animal species, and the cross-referencing of animal species with the different methods of analysis are described and summarized in both tables and figures. The reasons for the contradictions among different sources regarding the evaluated susceptibility of an animal species were also explored.

## Results

### Sources selected

After removal of duplicates, 3,306 and 6,266 sources were identified in the first and second rounds of source gathering, respectively. After the two sorting rounds and with the addition of sources through snowballing, expert recommendations, and the compilation of case reports from OIE, 97 sources were included in the scoping review (Figure 1).

### Characteristics of the included sources

Most sources were published or made available in 2020. There were 19 different countries in which the studies occurred, with China, then the USA, having the highest counts. There were nine different source types as self-described by the sources, the most common being journal articles. The number of animal species investigated per source ranged from 1 to over 300, with ⩽10 animal species investigated in most sources. *In silico* was the most common method used to evaluate a species susceptibility to SARS-CoV-2. Certain sources used multiple analysis methods; therefore, the total for this category does not equal 97 (Table 1).

**Table 1.** Characteristics of the literature sources selected for the scoping review.

Characteristics of studies	N (%)
Year	
2020	86 (88.66)
2021^†^	11 (11.34)
Country	
Australia	1 (1.03)
Bangladesh	1 (1.03)
Brazil	1 (1.03)
Canada	5 (5.15)
China	37 (38.14)
France	3 (3.09)
Germany	5 (5.15)
India	3 (3.09)
Iran	1 (1.03)
Italy	3 (3.09)
Japan	1 (1.03)
Malaysia	1 (1.03)
Mexico	1 (1.03)
Morocco	1 (1.03)
Netherlands	3 (3.09)
Republic of Korea	1 (1.03)
Spain	3 (3.09)
UK	4 (4.12)
USA	22 (22.68)
Source type (self-described by source)	
Communications	9 (9.28)
Correspondences	2 (2.06)
Dispatches	2 (2.06)
Essay and Perspectives	1 (1.03)
Journal articles	68 (70.10)
Letters	5 (5.15)
Preprints	8 (8.25)
Reports	1 (1.03)
Webpage	1 (1.03)
Study design^‡^	
*In silico*	46
*In vitro*	21
*In vivo*	36
Epidemiological	12
Number of animal species investigated per source	
⩽10	59 (60.82)
11-50	25 (25.77)
51-100	5 (5.15)
101-150	3 (3.09)
151-200	1 (1.03)
201-250	1 (1.03)
250-300	2 (2.06)
408	1 (1.03)

† For the year 2021, sources were collected up to April 30th.

‡ Total number does not equal 97 as some sources used more than one method of analysis.

### Results of individual sources of evidence

The full data charting table containing the author, title of source, date published/uploaded, source type, country of first author, susceptibility evaluating method, overview of the methods, number of animal species evaluated, and overview of findings for each source can be found in the attached Excel document, Supplemental Appendix S1. The animal species evaluated by each source, along with the taxonomic class and scientific and common names, can be found in the attached Excel document, Supplemental Appendix S2.

### Synthesis of results

#### Animal species evaluated

**Figure 2.**
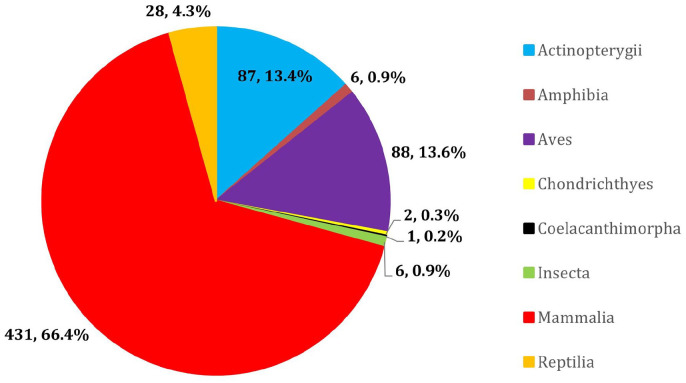
Total number of animal species (by taxonomic class) investigated in the sources chosen for the scoping review. A total of 649 animal species belonging to eight different classes were investigated by the 97 sources selected for the scoping review.

**Figure 3.**
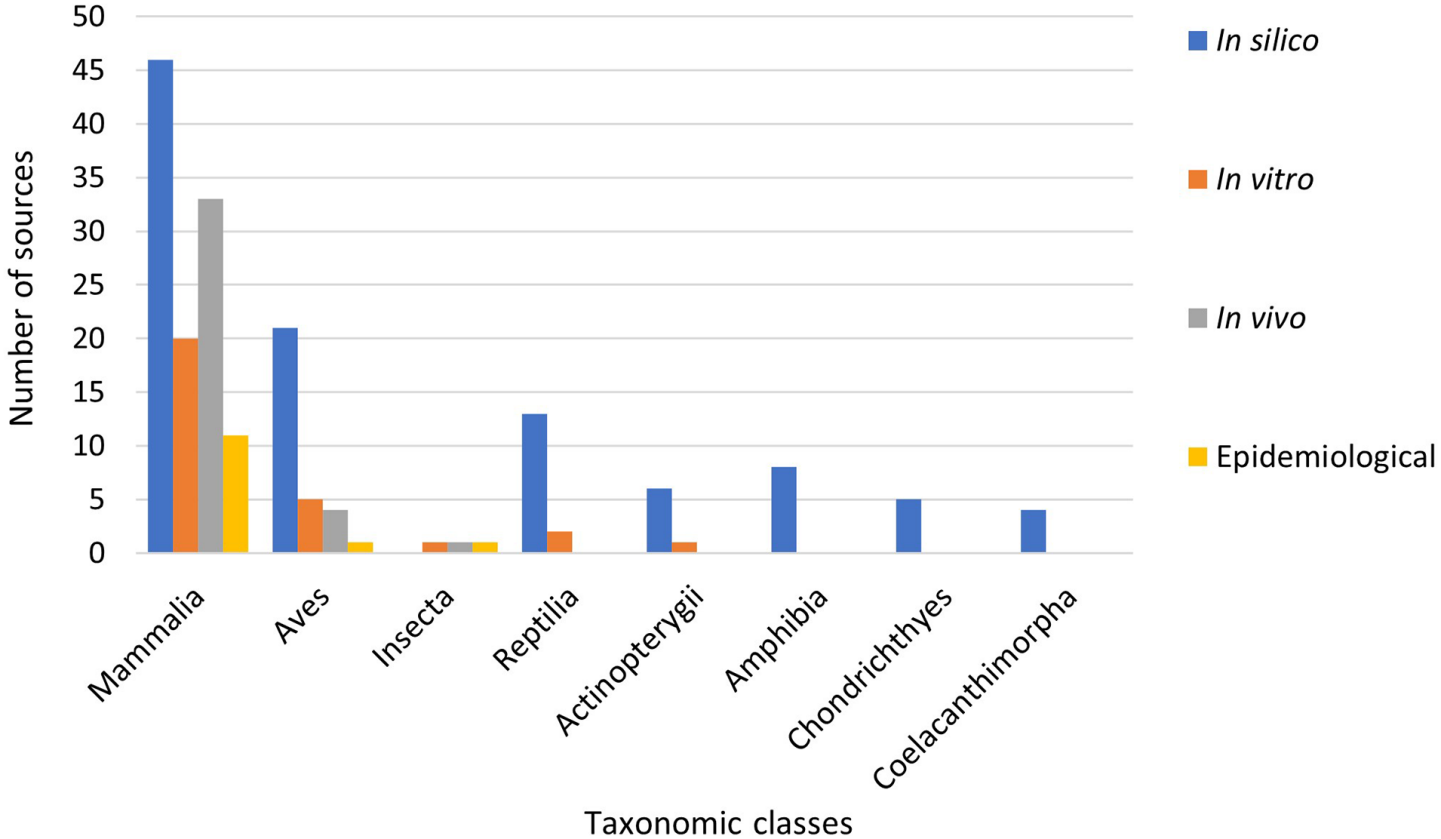
The number of sources identified in the scoping review that investigated each taxonomic class of animals for susceptibility to SARS-CoV-2, sorted by evaluation method. For each class, the number of sources along with the method used to determine susceptibility is shown. The corresponding numbers for the figure can be found in Table S1.”

Six hundred forty-nine animal species from eight classes were investigated in the 97 sources (Figure 2). Within the individual methods of evaluating susceptibility, mammalian species were the most studied class with 45 *in silico*, 20 *in vitro*, 33 *in vivo*, and 11 epidemiological studies. Aves was the second most investigated class in all methods except for epidemiological analysis, where there was a tie with Insecta. The *in silico* method investigated the most classes (n = 7) and was utilized by the most sources (Figure 3 and Supplemental Table S1).

In the total number of animal species investigated for each of the methods used to evaluate susceptibility, *in silico* dominated, investigating 633 out of the possible 649 species, followed by *in vitro* (129 species), epidemiological (42 species), and then *in vivo* (27 species). As a percentage for investigating the total number of each animal species in the different classes, the *in silico* method investigated 98% of the Mammalia, 99% of the Aves, and 100% of the Reptilia, Actinopterygii, Amphibia, Chondrichthyes, and Coelacanthimorpha species. Again, the Mammalia class had the most species investigated for each analysis method (Table 2).

**Table 2.** Total number of animal species investigated in the literature (based on taxonomic class) by each of the four susceptibility predicting methods.

Class	*In silico*	*In vitro*	*In vivo*	Epidemiological
Mammalia	422	118	19	35
Aves	87	4	5	5
Insecta	0	3	3	2
Reptilia	28	3	0	0
Actinopterygii	87	1	0	0
Amphibia	6	0	0	0
Chondrichthyes	2	0	0	0
Coelacanthimorpha	1	0	0	0
Total	633	129	27	42

#### Methods used to describe and evaluate susceptibility

How an animal species susceptibility to SARS-CoV-2 was evaluated varied among the different analysis methods, ultimately contributing to different meanings of “susceptibility” among the different sources.

For *in silico* analysis, an animal species’ susceptibility to SARS-CoV-2 was commonly evaluated through investigating the binding potential of an animal species ACE2 receptor to the SARS-CoV-2 RBD. By comparing the homology of the human ACE2 (hACE2) receptor to the ACE2 receptor of different animal species, binding potential could be assessed through: (1) evaluating the homology to the entire hACE2 sequence, (2) selecting critical residues utilized by the hACE2 receptor when binding to the SARS-CoV-2 RBD, (3) evaluating residues that are in close proximity and may alter binding, or (4) creating homology models where the hACE2 binding to the SARS-CoV-2 RBD was used as a template to model an animal species ACE2 receptor binding to the SARS-CoV-2 RBD. Based on the homology, susceptibility scores were created or, if the ACE2 residues of the animal species differed from the hACE2 critical residues, the effects of those mutations on binding could be explored.^13,16,20,21,26,45-69^ With homology modeling, the interactions between the ACE2 receptor and the SARS-CoV-2 RBD could be further examined through analyzing binding affinities, molecular dynamics, or docking simulations.^16,17,21,45-48,58,59,63,67,70-81^ Other *in silico* methods used to predict susceptibility include: (1) investigating the relative synonymous codon usage, which compares the codons of the viral genome to the codons used in different animal species; (2) comparing the homology of the human TMPRSS2 sequence to animal species; (3) creating statistical models or learning algorithms to predict susceptibility based on the characteristics of the ACE2 receptor, CoVs, or animal species; (4) investigating ACE2 isoforms and gene expression; or (5) comparing the ACE2 receptor sequence of different animal species.^7,9,21,47,51,57,67,71,73,82^

The methods used by *in vitro* analysis to evaluate and describe susceptibility investigated ACE2 receptor binding or cellular entry of SARS-CoV-2 in cell culture. Viral binding methods included expressing the ACE2 receptor of various animal species combined with the SARS-CoV-2 RBD expressed on cells or as an Fc fusion protein. Binding was determined through surface plasmon resonance, ELISA, flow cytometry, or immunofluorescence.^13,54,56,65,66,68,76,83^ Viral entry methods included expressing the ACE2 receptor of different animals on cells not permissive to SARS-CoV-2 entry, or infecting cell lines from animal species with a SARS-CoV-2 pseudo or live virus. Viral entry was determined by immunofluorescence, cytopathic effects, or isolation of viral RNA or infectious virus from the exposed cells.^13,14,17,54,61,65,68,69,76,83-91^ Finally, some *in vitro* methods investigated the location and concentration of an animal species ACE2 receptor or TMPRSS2 protease.^20,85,91^

*In vivo* methods demonstrated susceptibility to SARS-CoV-2 infection through the experimental exposure of an animal species, usually a mammal. Animal species were inoculated through various routes including intranasal, intratracheal, oral, aerosolization, ocular, or intragastric with doses of SARS-CoV-2 ranging from 10^2^ to 7 × 10^6^ TCID50 or 10^2^ to 1.1 × 10^6^ PFU. After an animal was inoculated, susceptibility to SARS-CoV-2 infection or disease was determined through the analysis of clinical signs, pathogenesis, detection of viral RNA, infectious virus, or antibodies, or direct or indirect contact transmission. For direct contact transmission, the inoculated animal was placed in the same cage or pen as a naïve animal, while for indirect contact, the inoculated animal and naïve animal were separated by a barrier although air was exchanged between the animals.^2,6,72,84,86-89,92-119^

Epidemiological studies involved evaluating domestic, zoo, or wild animals naturally exposed to SARS-CoV-2 for clinical signs, pathogenesis, viral RNA, infectious virus, antibodies, or transmission.^5,40,90,120-128^

For each method, any limitations specified by the authors were recorded (Supplemental Table S2).

#### Contrasting susceptibility evaluations

Contrasting results from the different methods used to evaluate an animal species susceptibility to SARS-CoV-2 were identified in the scoping review. Using the top six investigated species, *Felis catus* (cats), *Canis lupus familiaris* (dogs), *Sus scrofa* (pigs), *Mus musculus* (house mice), *Mustela putorius furo* (ferrets), and *Oryctolagus cuniculus* (European rabbits), the susceptibility of each species to SARS-CoV-2 as evaluated by the sources is listed and compared in Table 3. Results for *in silico* analysis had the most variability, whereas results for *in vivo* and epidemiological analysis were more consistent. The contrasting results were more prevalent in dogs and pigs, whereas susceptibility evaluations were more consistent for cats, house mice, and European rabbits.

**Table 3.** Evaluation of susceptibility for the top six animal species investigated as described by the selected sources, sorted by method of evaluation.

Species	Source ranking^†^	*In silico*	*In vitro*	*In vivo*	Epidemiological
Cats N = 47	Not Susceptible	N = 1			N = 1
	Very low susceptibility				N = 1
	Low susceptibility				
	Medium/Intermediate susceptibility	N = 3			
	Potentially susceptible	N = 4			
	Susceptible	N = 13 (6) ^†^	N = 1 (6)	N = 2	N = 8
	High susceptibility	N = 5		N = 2	
Dogs N = 39	Not Susceptible	N = 4	N = 1		N = 1
	Very low susceptibility			N = 1	N = 1
	Low susceptibility	N = 4		N = 1	
	Medium/Intermediate susceptibility	N = 1			
	Potentially susceptible	N = 3			
	Susceptible	N = 10 (6)	N = 1 (6)		N = 5
	High susceptibility				
Pigs N = 31	Not Susceptible	N = 4	N = 1 (2)	N = 1 (2)	N = 1
	Very low susceptibility				
	Low susceptibility	N = 2			
	Medium/Intermediate susceptibility	N = 1			
	Potentially susceptible	N = 2			
	Susceptible	N = 9 (5)	N = 2 (5)	N = 1	
	High susceptibility				
House mice N = 31	Not Susceptible	N = 14 (7)	N = 2 (7)		N = 1
	Very low susceptibility	N = 1			
	Low susceptibility	N = 5			
	Medium/Intermediate susceptibility				
	Potentially susceptible	N = 1			
	Susceptible				
	High susceptibility				
Ferrets N = 24	Not Susceptible	N = 1			N = 1
	Very low susceptibility	N = 1			
	Low susceptibility	N = 1			
	Medium/Intermediate susceptibility	N = 1			
	Potentially susceptible	N = 2			
	Susceptible	N = 9 (1)	N = 1 (2)	N = 2 (1)	N = 1
	High susceptibility	N = 1		N = 1	
European rabbits N = 24	Not Susceptible	N = 2			N = 1
	Very low susceptibility				
	Low susceptibility				
	Medium/Intermediate susceptibility	N = 1			
	Potentially susceptible	N = 1			
	Susceptible	N = 10 (5)	N = 1 (6)	N = (1)	
	High susceptibility	N = 2			

N refers to the number of sources. Sources that did not give a susceptibility classification were omitted from this table but can be found in Supplemental Appendix S1. References for Table 2 can be found in Supplemental Table S3.

† Numbers in parentheses represent sources that used more than one method of analysis and are shared between different analysis methods.

## Discussion

The literature on susceptibility of animal species to SARS-CoV-2 is growing at a rapid speed, reflecting the urgency of identifying animal reservoirs and potential animal models for vaccines and drug therapies. With many studies investigating various animal species using different methods, this scoping review identifies areas of consensus, including a focus on mammals (vs other classes of animals), as well as areas of and reasons for contrast, with different sources reporting different species’ susceptibility depending on methods and definitions. In addition, an early preponderance of studies relying on *in silico* methods, appropriate to early response, which served as useful guides to target species for further *in vivo* and epidemiological studies were identified.

### Source characteristics

Sources were uploaded or published either in 2020 or early 2021, as SARS-CoV-2 was detected in late 2019. Sources that were published or uploaded after the last round of source gathering were either expert recommendations or preprints which are now published.

Journal articles comprised most of the source types, which is reassuring, as peer-review presumably critically evaluated methodology and interpretation of results evaluating an animal’s susceptibility to SARS-CoV-2. However, with the novel nature of the pathogen, the high levels of uncertainty in the early pandemic, and the rapidly expanding literature on SARS-CoV-2, conflicting reports and disagreements between published articles are inevitable. For example, the paper by Ji et al^7^ which used relative synonymous codon usage, concluded snakes were possible intermediate hosts for SARS-CoV-2; however, this was refuted in subsequent papers.^129^

Sources originated from 19 different countries, reflecting the fact that SARS-CoV-2 is a global concern but also because different animal species are geographically bounded, requiring regional knowledge of fauna. China produced the greatest number of sources included in this review, most likely due to SARS-CoV-2 first being detected in China. In addition, CoV research was occurring in China before the global spread of the virus.

Most sources investigated 10 or fewer animal species; sources which investigated more than 10 primarily used *in silico* or *in vitro* analysis. These larger studies helped target species for more costly (in terms of time, resources, and animal use) investigations involving experimental infections, transmission, re-challenging, or necropsies.^130,131^ For example, early findings allowed researchers to target animals with a legitimate potential for successful infection (such as mammals), versus animals with little to low susceptibility (such as fish).

### Animal species investigated

Early *in silico* and *in vitro* findings steered investigation toward animal species belonging to class Mammalia, which is supported by subsequent findings that mammals have been successfully infected with SARS-CoV-2, both experimentally and naturally. Although unlikely, it is important to note that this bias might lead to missing some unusual potential animal hosts. Aves was the second most investigated class, and previous work has shown that Aves are commonly infected with delta and gamma CoVs. Although the CoVs that infect Mammalian and Aves species belong to different genera, exploring all avenues for susceptible animals, especially those known to be infected with CoVs, is essential.^10,132^ For the other classes investigated, the species were either chosen since they are classified as vertebrates and express the ACE2 receptor, or to test a specific purpose, such as if mosquitos could carry SARS-CoV-2.^61,90,101^

### Evaluating methods and animal species

The *in silico* method was employed the most and across the highest number of animal species and classes. This method is advantageous as it can cover a large swath of animal species in a relatively short period and at comparatively lower cost than other methods. Its efficiency demonstrates the utility of *in silico* methods to rapidly pre-screen numerous species, narrowing the focus on species and classes that are more likely to be susceptible for follow-up investigation using more resource-intensive methods.^133^ It is important to note that *in silico* results are not necessarily supported by the other methods. Encouragingly, as the results of *in vivo* and epidemiological analysis were published, many sources used these results to refine the accuracy of their *in silico* models.^60,73^

Somewhat surprisingly, more sources used *in vivo* versus *in vitro* methods, perhaps because this was thought to provide stronger evidence to determine animal models for SARS-CoV-2. Furthermore, many common laboratory animals were readily available (especially as non-SARS-CoV-2 research was paused) before *in vitro* cell lines could be made. The first *in vitro* study was available February 3rd, 2020, before any *in vivo* studies; then, prior to publication of the second *in vitro* study on May 13th, 2020, six *in vivo* studies became available.^2,14,72,91,106,108,115,117^ Furthermore, four *in vivo* studies investigating Syrian hamsters, a common lab animal, were available before the first *in vitro* study investigating Syrian hamsters.^20,72,102,105,110^ (Supplemental Appendix S1).

More species and classes were investigated using *in vitro* compared to *in vivo* methods. Thus, with *in vitro* methods, a greater diversity of species can be investigated, including the many potentially susceptible animal species that cannot be cultivated in the laboratory, such as cetaceans and large ungulates. Additionally, *in vitro* methods allow for investigation of species of high conservation concern.

Epidemiological studies in naturally exposed animals appeared less often due to the low occurrence of SARS-CoV-2 in domestic and wild animals in the early stages of the pandemic, and because OIE reports were combined into one source. The number of species investigated in epidemiological studies, however, was higher than *in vivo*. This is largely due to the impact of a single source, Deng et al^121^ which investigated serological response in 35 potentially naturally exposed animal species; if removed, only 13 animal species would have been investigated. This may also reflect lag times in securing animal research ethics approval for experimental exposure of captive animals, and responsible animal use.

### Variations among studies evaluating susceptibility

The term “susceptibility” was used variably depending on the methods used. For *in silico* and *in vitro* analysis, susceptibility meant that animal species potentially could, or have, the capacity to become infected, with SARS-CoV-2. Whereas for *in vivo* and epidemiological analysis, susceptible hosts were those in which the virus can replicate and transmit to other hosts. These differences demonstrate how susceptibility can be a subjective term, possibly resulting in misunderstandings when interpreting the results if the audience is unfamiliar with the capabilities of each method.

Depending on the species, sources reported different results for susceptibility to SARS-CoV-2, even when using similar methods, this was evident for both dogs and pigs.

Overall, *in silico* analysis had the most variable susceptibility evaluations among the different analysis methods, followed by *in vitro* analysis. *In vivo* and epidemiological analysis were more consistent in their susceptibility evaluations. For *in silico*, the variance in susceptibility predictions were in part due to the ranging methods used to predict susceptibility, from comparing certain hACE2 critical residues to the ACE2 residues of select animals to more in-depth analysis such as homology modeling with follow up analysis including binding affinities or docking simulations. In addition, simulated modeling and the infection of a single cell may not translate to the real world, where additional characteristics will impact whether an animal becomes infected or ill, and/or is capable of transmission.^130,133,134^ These additional characteristics include the concentration and location of the ACE2 receptor, viral avoidance of host immune response, the potential for ACE2 isoforms that inhibit cellular entry, and/or the acquisition of cellular components for replication.^20,51,52,59,65,75^ If SARS-CoV-2 fails in any of these regards, chances of an established infection decrease, which demonstrates the importance of follow-up *in vivo* and epidemiological analyses.

Differences in susceptibility derived from experimental infection through *in vivo* studies and natural infection in epidemiological studies also require careful interpretation. Results from *in vivo* testing are dependent on the dose, route of inoculation, and monitoring indicators such as detectable viral RNA, infectious virus, and antibodies.^130,131^ If conspecific animals receive different doses of SARS-CoV-2, and the animal with the higher dose is deemed infected but the animal that received the lower dose is negative, whether the animal species should be considered susceptible under natural circumstances depends greatly on how closely the experimental conditions mimic natural transmission and infective doses. In pigs inoculated with 1 × 10^5^ or 1 × 10^6^ TCID, three sources determined pigs were not susceptible, while the fourth determined pigs to be susceptible based on observation of ocular discharge, detection of viral RNA from nasal washes in two pigs and a communal chew rope, recovery of infectious virus from a submandibular lymph node in one pig, and detection of neutralizing antibodies in two other pigs.^2,86,89,119^

In both *in vivo* and epidemiological studies, interpretation of susceptibility should also consider the indicators used to determine infection status: that is antibodies, detection of viral RNA, recovery of live virus, transmission, and the timeframe. Virus or RNA is detected in animals before antibodies are present. Conversely, detection of antibodies does not necessarily equate to the animal being truly infected or competent for transmission, only that the animal was previously exposed to SARS-CoV-2.^135^ Therefore, detection of viral RNA and, especially, infectious virus are more definitive indicators of infection status; however, there may be biosafety reasons why recovery of live virus is not feasible. Assessing transmission is also valuable as it shows that an animal species cannot only become infected but also infect other animals, making it an ideal intermediate and possible reservoir host.^136^ In dogs, the contrasting susceptibility predictions between *in vivo* and epidemiological analysis stems from epidemiological analysis determining dogs were susceptible through the detection of antibodies or viral RNA, while *in vivo* analysis, which used more specific indicators for SARS-CoV-2 susceptibility, such as transmission, determined dogs had a lower susceptibility.^2,5,40,92,120,122,124^ The latter is also borne out by observations that dogs only rarely become infected or ill with SARS-CoV-2, generally in households with close, prolonged contact with infected people.^40,127,137^

The genetics of the animal can also affect the outcome. Most laboratory strains of animals are genetically engineered, pathogen free, and kept in artificial husbandry conditions, which does not mimic the real world, where domestic and wild animals are genetically diverse, may experience nutritional stress, and are subject to a barrage of other pathogens.^131,138^ Epidemiological analyses of domestic animals should also consider animal co-morbidities (chronic disease, immunosuppression) as we have observed in human populations, where severe disease associated with SARS-CoV-2 is frequently linked to other risk factors.^131,138^

## Conclusions and Future Work

For the different methods used to evaluate an animal’s susceptibility to SARS-CoV-2 (and other emerging zoonoses), it would be optimal to use *in silico* and *in vitro* to screen multiple animal species in a rapid and inexpensive fashion early in a pandemic, followed by *in vivo* or epidemiological analysis, with a preference for detecting infectious virus and/or viral RNA. Antibody testing could also be used as a secondary screening tool to prioritize animal species to determine reservoir and bridging hosts for SARS-CoV-2. This integrated approach has demonstrated success in different areas of research including toxicology and virulence.^130,139-141^

Based on the results from the sources included in this scoping review, susceptible mammals with a peridomestic or commensal relationship with humans could be closely monitored as a potential reservoir species.^142,143^ Although not an exhaustive list, species that could be monitored are found within the mustelid, cricetid, and cervid families. Ferrets and minks (mustelids), have both demonstrated a high susceptibility to SARS-CoV-2 infection through *in vivo* and epidemiological analysis.^2,28,40,89,117,144-146^ Also, in the USA and Italy, viral RNA was detected in wild minks, and in a pet ferret.^40,144,145^ Deer mice, Syrian hamsters, and dwarf hamsters, in the cricetid family, have shown high susceptibility through *in vivo* analysis (infectious virus, viral RNA, antibodies, and transmission detected).^95,98,102,105,147^ Although not susceptible to the initial SARS-CoV-2 variant, Old World rodent species have demonstrated increased susceptibility to SARS-CoV-2 variants.^148^ White-tailed deer (cervids) were experimentally infected with SARS-CoV-2. Viral RNA, infectious virus, antibodies, and transmission were subsequently detected. Epidemiological analysis also revealed antibodies in 40% of tested wild deer in the USA, indicating some form of natural exposure.^88,149^

Next steps could include further scoping reviews with up-to-date sources, conducting systematic reviews where the different methods of evaluating susceptibility are evaluated and ranked, and/or meta-analyses for combining the results of select animal species based on their evaluated susceptibility. Of the species determined to be susceptible from *in vivo* methods, assessing them for natural exposure is a critical next step in determining their potential to become reservoir species, increasingly important as the pandemic becomes better managed in humans and the rise of variants threatens the efficacy of existing diagnostic assays and vaccinations. The breadth of information surrounding an animal species’ susceptibility to SARS-CoV-2 is extensive and increasing. This scoping review demonstrated the utility and limitations of the rapidly expanding (and often overwhelming) literature evaluating susceptibility of animals to an emerging, global zoonoses, which can be helpful in planning and surveillance in the existing pandemic, and in preparing for future emerging disease events.

The limitations for this scoping review include the exclusion of non-English sources and missing relevant sources due to the sheer volume of literature. Moreover, as the last search for sources occurred in January 2021, there are likely new sources available that include animal species not presently included in this scoping review. Even with these limitations, this scoping review is important for those designing studies to determine animal susceptibility to a novel pathogen, and to efficiently target surveillance for potential animal reservoirs for SARS-CoV-2.

## Supplemental Material

sj-docx-1-ehi-10.1177_11786302221107786 – Supplemental material for Investigating SARS-CoV-2 Susceptibility in Animal Species: A Scoping Review Click here for additional data file. Supplemental material, sj-docx-1-ehi-10.1177_11786302221107786 for Investigating SARS-CoV-2 Susceptibility in Animal Species: A Scoping Review by Connor Rutherford, Pratap Kafle, Catherine Soos, Tasha Epp, Lori Bradford and Emily Jenkins in Environmental Health Insights

sj-docx-2-ehi-10.1177_11786302221107786 – Supplemental material for Investigating SARS-CoV-2 Susceptibility in Animal Species: A Scoping Review Click here for additional data file. Supplemental material, sj-docx-2-ehi-10.1177_11786302221107786 for Investigating SARS-CoV-2 Susceptibility in Animal Species: A Scoping Review by Connor Rutherford, Pratap Kafle, Catherine Soos, Tasha Epp, Lori Bradford and Emily Jenkins in Environmental Health Insights

sj-jpg-4-ehi-10.1177_11786302221107786 – Supplemental material for Investigating SARS-CoV-2 Susceptibility in Animal Species: A Scoping Review Click here for additional data file. Supplemental material, sj-jpg-4-ehi-10.1177_11786302221107786 for Investigating SARS-CoV-2 Susceptibility in Animal Species: A Scoping Review by Connor Rutherford, Pratap Kafle, Catherine Soos, Tasha Epp, Lori Bradford and Emily Jenkins in Environmental Health Insights

sj-xlsx-3-ehi-10.1177_11786302221107786 – Supplemental material for Investigating SARS-CoV-2 Susceptibility in Animal Species: A Scoping Review Click here for additional data file. Supplemental material, sj-xlsx-3-ehi-10.1177_11786302221107786 for Investigating SARS-CoV-2 Susceptibility in Animal Species: A Scoping Review by Connor Rutherford, Pratap Kafle, Catherine Soos, Tasha Epp, Lori Bradford and Emily Jenkins in Environmental Health Insights
