# Supplementary material for: Investigating SARS-CoV-2 Susceptibility in Animal Species: A Scoping Review
Source: Environ Health Insights. 2022 Jun 28;16:11786302221107786. doi: 10.1177/11786302221107786 (PMC9247998; doi:10.1177/11786302221107786)
Supplement: sj-docx-2-ehi-10.1177_11786302221107786 – Supplemental material for Investigating SARS-CoV-2 Susceptibility in Animal Species: A Scoping Review [file sj-docx-2-ehi-10.1177_11786302221107786.docx]

**Supplementary Figures, Tables, and Appendices**

[Insert Figure S1.]

Literature sources were gathered from databases from July 9^th^-13^th^ 2020 and December 30^th^- January 2^nd^, 2021. Above are the search terms used for the Public Health Database.

**Table S1.** The number of sources that investigated each taxonomic class sorted by susceptibility evaluating method (see Figure 4).

| **Class** | ***In silico*** | ***In vitro*** | ***In vivo*** | **Epidemiological** |
| --- | --- | --- | --- | --- |
| **Mammalia** | 46 | 20 | 33 | 11 |
| **Aves** | 21 | 5 | 4 | 1 |
| **Insecta** | 0 | 1 | 1 | 1 |
| **Reptilia** | 13 | 2 | 0 | 0 |
| **Actinopterygii** | 6 | 1 | 0 | 0 |
| **Amphibia** | 8 | 0 | 0 | 0 |
| **Chondrichthyes** | 5 | 0 | 0 | 0 |
| **Coelacanthimorpha** | 4 | 0 | 0 | 0 |

**Table S2.** Self-reported limitations from the sources selected for the scoping review, sorted by analysis method.

| **Limitations described by sources** | **References** |
| --- | --- |
| ***In silico*** |  |
| Susceptibility and risk were generalized to an entire species however intraspecies variation may exist in the ACE2 sequence which could alter a subspecies susceptibility | 1–5 |
| ACE2 isoforms may be present which could alter an animals predicted susceptibility | 6 |
| Only mammal’s species used which had phylogenetic, ecological, and geospatial data available | 7 |
| More species should have been evaluated, however limited due to missing information, ACE2 receptors not found in databases, or phylogenetic, ecological, and geospatial data was unavailable | 4,5,7–9 |
| Majority of animal species only selected which were known to be infected with CoVs | 7 |
| Only RNA expression of ACE2 investigated did not consider protein expression | 6 |
| More to an animal’s susceptibility than ACE2 expression | 6 |
| ACE2 expression levels were unknown | 10 |
| The crystal structure, the protein sequence, and what effects expression of TMPRSS2 is unknown which could impact susceptibility | 10 |
| Possible that other receptors besides ACE2 or regions on the ACE2 receptor are used by SARS-CoV-2 therefore susceptibility predictions may not be accurate | 3,11,12 |
| There is more to an animal being susceptible besides the interaction between the SARS-CoV-2 RBD and the ACE2 receptor other factors that need to be considered, host immune response, factors which would allow virus to prosper, underlying health conditions, host behavior and number of contacts, age, atmospheric temperature, population density, airflow, ventilation, and humidity | 2,3,5,12–19 |
| Predictions based on the homology to the hACE2 receptor may under or overestimate the impact of single mutations, glycosylated residues, or other factors which could increase or reduce binding | 1,2,5,8,18 |
| That predictions should be further verified using additional information or methods like experimental infection, epidemiological investigations, biochemical or biophysical approaches | 1,5,8,12–15,20–26 |
| More data on how the mutations on the ACE2 receptors will impact binding | 12 |
| Different crystal structures of the hACE2:SARS-CoV-2 RBD used to model the ACE2:SARS-CoV-2 RBD gave different binding results | 27 |
| Only used CoVs with full length genomes used | 7 |
| How only one SARS-CoV-2 strain was used, and other strains or variants may have a different outcome | 3,15 |
| ***In vitro*** |  |
| Species specific cell lines were not used | 28,29 |
| Possibility that other receptors besides ACE2 can be used | 28 |
| Binding and or entry does not equate to susceptibility additional factors that can impact an animal’s susceptibility include entry after binding, other cellular factors, transmission of the virus | 18,28–30 |
| ACE2 polymorphisms present in an animal which can give conflicting results | 31 |
| Experimental infections or epidemiology analysis needed to further determine intermediate hosts | 32,33 |
| A pseudotyped virus system is limited in scope and does not allow for further experimentation | 34 |
| Only assessed ACE2 functionalities for determining host range other factors might also contribute | 18 |
| ACE2 sequences obtained from a database no experimental evidence that these genes can code a functional protein | 18 |
| Accurate predictions are difficult due to the lack of animal infection data and biochemical interactions between the SARS-CoV-2 RBD and ACE2 receptor of different animals | 24 |
| ***In vivo*** |  |
| Small sample size | 35,36(p2),37,38 |
| Experimental animals were young, healthy, and/or immunocompetent, animals naturally infected may respond differently to infection possibly having increased susceptibility, severity, transmission, or shedding | 37,39–46 |
| Differences in age, breed, and colony of animals between experimental studies can affect outcomes between studies | 38 |
| Comparison between rechallenged and primary challenged not conducted therefore unable to determine if the results from the rechallenged animals are from the primary or rechallenged infection | 47 |
| Inoculum doses differed or other doses needed to fully describe and understand patterns in pathogenesis | 35,38,48,49 |
| Variants or other strains can alter pathogenesis and susceptibility | 38,43,49 |
| Limited as an animal model as infection not fully replicated, no severe disease present, infectious virus not found, reinfection studies not conducted, or treatment group was not included | 37,39,44,50–53 |
| Physiological body temperatures was not known prior to the study | 42,44 |
| Chemokines or cytokines expression were investigated partially or not at all | 44,48,54 |
| No positive control samples | 55 |
| Only SARS-CoV-2 spike gene sequences instead of whole genome | 48 |
| Could not determine transmission pathway aerosol, droplet, or both | 56 |
| Usure how transmission studies will relate to the real world including both animal to human and animal to animal | 40 |
| Pathology between animals could not be compared as animals sacrificed at different times and were outbred | 44 |
| Unsure how infection was established through oral inoculation | 51 |
| Unable to determine what areas of the immune system provided protection from reinfection | 50 |
| **Epidemiological** |  |
| Small sample size | 57–60 |
| Experimental infections needed to further determine susceptibility | 61,62 |
| Neuter status unknown prevents better comparison with humans | 58 |
| Detection of viral RNA from oropharyngeal swab may have been due to cat coming into contact with viral RNA instead of viral infection | 59 |
| Cannot determine if virus actively replicated as no culture assays conducted | 59 |
| A focus should be on different ages and different degrees of viral load | 60 |
| Unable to estimate time of infection in the animals | 58 |
| Could only determine exposure could not determine naturally if dogs and cats can transmit or become infected with SARS-CoV-2 | 63 |
| Missing SARS-CoV-2 infection status of pet owners | 64 |

**Table S3.** Sources that evaluated and described the susceptibility to SARS-CoV-2 of the six most investigated animal species from the scoping review, by evaluation method.

|  | **Source ranking** | ***In silico*** | ***In vitro*** | ***In vivo*** | **Epidemiological** |
| --- | --- | --- | --- | --- | --- |
| **Cats N=47** | **Not Susceptible** | 65 |  |  | 61 |
|  | **Very low susceptibility** |  |  |  | 60 |
|  | **Medium / Intermediate susceptibility** | 4,12,66 |  |  |  |
|  | **Potentially susceptible** | 11,23,67,68 |  |  |  |
|  | **Susceptible** | 6,7,9,14,15,17–19,21,26,27,29,32,34,69–73 | 18,29,30,32,34,72,73 | 39,74 | 57–59,63,64,75–77 |
|  | **Highly susceptible** | 1,10,22,25,78 |  | 79,80 |  |
| **Dogs N=39** | **Not Susceptible** | 4,65,70,71 |  | 81 | 61 |
|  | **Very low susceptibility** |  |  | 80 | 60 |
|  | **Low susceptibility** | 12,22,66,78 |  | 79 |  |
|  | **Medium / Intermediate susceptibility** | 10 |  |  |  |
|  | **Potentially susceptible** | 11,23,68 |  |  |  |
|  | **Susceptible** | 1,6,7,9,14,15,18,19,21,27,29,32,34,69,73,82 | 18,29,30,32,34,73,82 |  | 57,58,63,75,76 |
| **Pigs N=31** | **Not Susceptible** | 19,27,65,71 | 33,37,43 | 37,43,79 | 61 |
|  | **Low susceptibility** | 12,22 |  |  |  |
|  | **Medium / Intermediate susceptibility** | 10 |  |  |  |
|  | **Potentially susceptible** | 9,11 |  |  |  |
|  | **Susceptible** | 1,7,14,15,17,18,21,26,29,32,66,69,73,82 | 18,29,30,32,73,82,83 | 38 |  |
| **House Mice N=31** | **Not Susceptible** | 6,7,9–11,14,15,17,18,21,23,26,27,29,32,34,65,68,72,73,82 | 18,29,30,32,34,72,73,82,83 |  | 61 |
|  | **Very low susceptibility** | 12 |  |  |  |
|  | **Low susceptibility** | 1,19,22,25,66 |  |  |  |
|  | **Potentially susceptible** | 67 |  |  |  |
| **Ferrets N=24** | **Not Susceptible** | 65 |  |  | 61 |
|  | **Very low susceptibility** | 12 |  |  |  |
|  | **Low susceptibility** | 66 |  |  |  |
|  | **Medium / Intermediate susceptibility** | 10 |  |  |  |
|  | **Potentially susceptible** | 9,11 |  |  |  |
|  | **Susceptible** | 6,14,15,17–19,21,23,27,71 | 18,30,37 | 37,52,56 | 76 |
|  | **Highly susceptible** | 22 |  | 79 |  |
| **European rabbits N=24** | **Not Susceptible** | 23,65 |  |  | 61 |
|  | **Very low susceptibility** |  |  |  |  |
|  | **Low susceptibility** |  |  |  |  |
|  | **Medium / Intermediate susceptibility** | 12 |  |  |  |
|  | **Potentially susceptible** | 9 |  |  |  |
|  | **Susceptible** | 7,14–16,18,19,21,26,32,34,69–73 | 18,30,32,34,46,72,73 | 46 |  |
|  | **Highly susceptible** | 1,22 |  |  |  |

**Appendix S1.** Data charting for the scoping review.

Contains the individual characteristics of the sources selected for the scoping review. The characteristics of each source include: the first three authors, source title, date uploaded / published, document type, country of first author, susceptibility evaluating method, an overview of the methods used, number of animal species investigated, and an overview of the findings.

**Appendix S2.** Animal species investigated by the sources.

Contains the animal species investigated by each source. For each animal species, their class, scientific, and common name are listed. For each source, the first three authors are listed.

**References**

1. Ahmed R, Hasan R, Siddiki AMAMZ, Islam MS. Host range projection of SARS-CoV-2: South Asia perspective. *Infect Genet Evol*. 2021;87:104670. doi:10.1016/j.meegid.2020.104670

2. Frank HK, Enard D, Boyd SD. Exceptional diversity and selection pressure on SARS-CoV and SARS-CoV-2 host receptor in bats compared to other mammals. *bioRxiv*. Published online April 20, 2020:2020.04.20.051656. doi:10.1101/2020.04.20.051656

3. Martínez-Hernández F, Isaak-Delgado AB, Alfonso-Toledo JA, et al. Assessing the SARS-CoV-2 threat to wildlife: potential risk to a broad range of mammals. *Perspect Ecol Conserv*. 2020;18(4):223-234. doi:10.1016/j.pecon.2020.09.008

4. Mathavarajah S, Stoddart AK, Gagnon GA, Dellaire G. Pandemic danger to the deep: the risk of marine mammals contracting SARS-CoV-2 from wastewater. *Sci Total Environ*. 2021;760:143346. doi:10.1016/j.scitotenv.2020.143346

5. Melin AD, Janiak MC, Marrone F, Arora PS, Higham JP. Comparative ACE2 variation and primate COVID-19 risk. *Commun Biol*. 2020;3(1):641. doi:10.1038/s42003-020-01370-w

6. Sun K, Gu L, Ma L, Duan Y. Atlas of ACE2 gene expression reveals novel insights into transmission of SARS-CoV-2. *Heliyon*. 2021;7(1):e05850. doi:10.1016/j.heliyon.2020.e05850

7. Wardeh M, Baylis M, Blagrove MSC. Predicting mammalian hosts in which novel coronaviruses can be generated. *Nat Commun*. 2021;12:780. doi:10.1038/s41467-021-21034-5

8. Kumar A, Pandey SN, Pareek V, Narayan RK, Faiq MA, Kumari C. Predicting susceptibility for SARS-CoV-2 infection in domestic and wildlife animals using ACE2 protein sequence homology. *Zoo Biol*. 2021;40(1):79-85. doi:10.1002/zoo.21576

9. Luan J, Lu Y, Jin X, Zhang L. Spike protein recognition of mammalian ACE2 predicts the host range and an optimized ACE2 for SARS-CoV-2 infection. *Biochem Biophys Res Commun*. 2020;526(1):165-169. doi:10.1016/j.bbrc.2020.03.047

10. Alexander MR, Schoeder CT, Brown JA, et al. Predicting susceptibility to SARS-CoV-2 infection based on structural differences in ACE2 across species. *The FASEB Journal*. 2020;34(12):15946-15960. doi:10.1096/fj.202001808R

11. Li R, Qiao S, Zhang G. Analysis of angiotensin-converting enzyme 2 (ACE2) from different species sheds some light on cross-species receptor usage of a novel coronavirus 2019-nCoV. *J Infect*. 2020;80(4):469-496. doi:10.1016/j.jinf.2020.02.013

12. Damas J, Hughes GM, Keough KC, et al. Broad host range of SARS-CoV-2 predicted by comparative and structural analysis of ACE2 in vertebrates. *PNAS*. 2020;117(36):22311-22322. doi:10.1073/pnas.2010146117

13. Zhai X, Sun J, Yan Z, et al. Comparison of SARS-CoV-2 spike protein binding to ACE2 receptors from human, pets, farm animals, and putative intermediate hosts. *J Virol*. 2020;94(15):e00831-20. doi:10.1128/JVI.00831-20

14. Huang X, Zhang C, Pearce R, Omenn GS, Zhang Y. Identifying the zoonotic origin of SARS-CoV-2 by modeling the binding affinity between the spike receptor-binding domain and host ACE2. *J Proteome Res*. 2020;19(12):4844-4856. doi:10.1021/acs.jproteome.0c00717

15. Lam SD, Bordin N, Waman VP, et al. SARS-CoV-2 spike protein predicted to form complexes with host receptor protein orthologues from a broad range of mammals. *Sci Rep*. 2020;10(1):16471. doi:10.1038/s41598-020-71936-5

16. Preziuso S. *Severe acute respiratory syndrome coronavirus* 2 (SARS-CoV-2) exhibits high predicted binding affinity to ACE2 from Lagomorphs (rabbits and pikas). *Animals (Basel)*. 2020;10(9):1460. doi:10.3390/ani10091460

17. Wan Y, Shang J, Graham R, Baric RS, Li F. Receptor recognition by the novel coronavirus from Wuhan: an analysis based on decade-long structural studies of SARS coronavirus. *J Virol*. 2020;94(7):e00127-20. doi:10.1128/JVI.00127-20

18. Liu Y, Hu G, Wang Y, et al. Functional and genetic analysis of viral receptor ACE2 orthologs reveals a broad potential host range of SARS-CoV-2. *PNAS*. 2021;118(12):e2025373118. doi:10.1073/pnas.2025373118

19. Praharaj MR, Garg P, Kesarwani V, et al. SARS-CoV-2 spike glycoprotein and ACE2 interaction reveals modulation of viral entry in wild and domestic animals. *bioRxiv*. Published online January 8, 2021:2020.05.08.084327. doi:10.1101/2020.05.08.084327

20. Ji W, Wang W, Zhao X, Zai J, Li X. Cross-species transmission of the newly identified coronavirus 2019-nCoV. *J Med Virol*. 2020;92(4):433-440. doi:10.1002/jmv.25682

21. Luan J, Jin X, Lu Y, Zhang L. SARS‐CoV‐2 spike protein favors ACE2 from Bovidae and Cricetidae. *J Med Virol*. Published online April 10, 2020. doi:10.1002/jmv.25817

22. Sang ER, Tian Y, Gong Y, Miller LC, Sang Y. Integrate structural analysis, isoform diversity, and interferon-inductive propensity of ACE2 to predict SARS-CoV2 susceptibility in vertebrates. *Heliyon*. 2020;6(9):e04818. doi:10.1016/j.heliyon.2020.e04818

23. Brooke GN, Prischi F. Structural and functional modelling of SARS-CoV-2 entry in animal models. *Sci Rep*. 2020;10:15917. doi:10.1038/s41598-020-72528-z

24. Low-Gan J, Huang R, Warner G, Kelley A, McGregor D, Smider V. Diversity of ACE2 and its interaction with SARS-CoV-2 receptor binding domain. *bioRxiv*. Published online November 4, 2020:2020.10.25.354548. doi:10.1101/2020.10.25.354548

25. Shen M, Liu C, Xu R, et al. Predicting the animal susceptibility and therapeutic drugs to SARS-CoV-2 based on spike glycoprotein combined with ACE2. *Front Genet*. 2020;11:575012. doi:10.3389/fgene.2020.575012

26. Qiu Y, Zhao YB, Wang Q, et al. Predicting the angiotensin converting enzyme 2 (ACE2) utilizing capability as the receptor of SARS-CoV-2. *Microbes Infect*. 2020;22(4-5):221-225. doi:10.1016/j.micinf.2020.03.003

27. Delgado Blanco J, Hernandez-Alias X, Cianferoni D, Serrano L. *In silico* mutagenesis of human ACE2 with S protein and translational efficiency explain SARS-CoV-2 infectivity in different species. *PLoS Comput Biol*. 2020;16(12):e1008450. doi:10.1371/journal.pcbi.1008450

28. Yan H, Jiao H, Liu Q, et al. ACE2 receptor usage reveals variation in susceptibility to SARS-CoV and SARS-CoV-2 infection among bat species. *Nat Ecol Evol*. 2021;5(5):600-608. doi:10.1038/s41559-021-01407-1

29. Zhang HL, Li YM, Sun J, et al. Evaluating angiotensin-converting enzyme 2-mediated SARS-CoV-2 entry across species. *J Biol Chem*. 2021;296:100435. doi:10.1016/j.jbc.2021.100435

30. Conceicao C, Thakur N, Human S, et al. The SARS-CoV-2 spike protein has a broad tropism for mammalian ACE2 proteins. *PLoS Biol*. 2020;18(12):e3001016. doi:10.1371/journal.pbio.3001016

31. Liu K, Tan S, Niu S, et al. Cross-species recognition of SARS-CoV-2 to bat ACE2. *PNAS*. 2021;118(1):e2020216118. doi:10.1073/pnas.2020216118

32. Wu L, Chen Q, Liu K, et al. Broad host range of SARS-CoV-2 and the molecular basis for SARS-CoV-2 binding to cat ACE2. *Cell Discov*. 2020;6(68):1-12. doi:10.1038/s41421-020-00210-9

33. Di Teodoro G, Valleriani F, Puglia I, et al. SARS-CoV-2 replicates in respiratory ex vivo organ cultures of domestic ruminant species. *Vet Microbiol*. 2021;252:108933. doi:10.1016/j.vetmic.2020.108933

34. Zhao X, Chen D, Szabla R, et al. Broad and differential animal angiotensin-converting enzyme 2 receptor usage by SARS-CoV-2. *J Virol*. 2020;94(18):e00940-20. doi:10.1128/JVI.00940-20

35. Deng W, Bao L, Gao H, et al. Ocular conjunctival inoculation of SARS-CoV-2 can cause mild COVID-19 in rhesus macaques. *Nat Commun*. 2020;11(1):4400. doi:10.1038/s41467-020-18149-6

36. Munster VJ, Feldmann F, Williamson BN, et al. Respiratory disease in rhesus macaques inoculated with SARS-CoV-2. *Nature*. 2020;585(7824):268-272. doi:10.1038/s41586-020-2324-7

37. Schlottau K, Rissmann M, Graaf A, et al. SARS-CoV-2 in fruit bats, ferrets, pigs, and chickens: an experimental transmission study. *Lancet Microbe*. 2020;1(5):e218-e225. doi:10.1016/S2666-5247(20)30089-6

38. Pickering BS, Smith G, Pinette MM, et al. Susceptibility of domestic swine to experimental infection with severe acute respiratory syndrome coronavirus 2. *Emerg Infect Dis*. 2021;27(1):104-112. doi:10.3201/eid2701.203399

39. Gaudreault NN, Trujillo JD, Carossino M, et al. SARS-CoV-2 infection, disease and transmission in domestic cats. *Emerg Microbes Infect*. 2020;9(1):2322-2332. doi:10.1080/22221751.2020.1833687

40. Griffin BD, Chan M, Tailor N, et al. North American deer mice are susceptible to SARS-CoV-2. *bioRxiv*. Published online July 26, 2020:2020.07.25.221291. doi:10.1101/2020.07.25.221291

41. Hartman AL, Nambulli S, McMillen CM, et al. SARS-CoV-2 infection of African green monkeys results in mild respiratory disease discernible by PET/CT imaging and shedding of infectious virus from both respiratory and gastrointestinal tracts. *PLoS Pathog*. 2020;16(9):e1008903. doi:10.1371/journal.ppat.1008903

42. Lu S, Zhao Y, Yu W, et al. Comparison of SARS-CoV-2 infections among 3 species of non-human primates. *bioRxiv*. Published online June 17, 2020:2020.04.08.031807. doi:10.1101/2020.04.08.031807

43. Meekins DA, Morozov I, Trujillo JD, et al. Susceptibility of swine cells and domestic pigs to SARS-CoV-2. *Emerg Microbes Infect*. 2020;9(1):2278-2288. doi:10.1080/22221751.2020.1831405

44. Xu L, Yu DD, Ma YH, et al. COVID-19-like symptoms observed in Chinese tree shrews infected with SARS-CoV-2. *Zool Res*. 2020;41(5):517-526. doi:10.24272/j.issn.2095-8137.2020.053

45. Francisco R, Hernandez SM, Mead DG, et al. Experimental susceptibility of North American raccoons (*Procyon lotor*) and striped skunks (*Mephitis mephitis*) to SARS-CoV-2. *bioRxiv*. Published online March 8, 2021:2021.03.06.434226. doi:10.1101/2021.03.06.434226

46. Mykytyn AZ, Lamers MM, Okba NMA, et al. Susceptibility of rabbits to SARS-CoV-2. *Emerg Microbes Infect*. 2021;10(1):1-7. doi:10.1080/22221751.2020.1868951

47. Woolsey C, Viktoriya B, Prasad AN, et al. Establishment of an African green monkey model for COVID-19 and protection against re-infection. *Nat Immunol*. 2021;22(1):86-98. doi:10.1038/s41590-020-00835-8

48. Chan JFW, Zhang AJ, Yuan S, et al. Simulation of the clinical and pathological manifestations of Coronavirus Disease 2019 (COVID-19) in golden syrian hamster model: implications for disease pathogenesis and transmissibility. *Clin Infect Dis*. 2020;71(9):2428-2446. doi:10.1093/cid/ciaa325

49. Fagre A, Lewis J, Eckley M, et al. SARS-CoV-2 infection, neuropathogenesis and transmission among deer mice: Implications for reverse zoonosis to New World rodents. *bioRxiv*. Published online August 7, 2020:2020.08.07.241810. doi:10.1101/2020.08.07.241810

50. Chandrashekar A, Liu J, Martinot AJ, et al. SARS-CoV-2 infection protects against rechallenge in rhesus macaques. *Science*. 2020;369(6505):812-817. doi:10.1126/science.abc4776

51. Lee ACY, Zhang AJ, Chan JFW, et al. Oral SARS-CoV-2 inoculation establishes subclinical respiratory infection with virus shedding in golden syrian hamsters. *Cell Rep Med*. 2020;1(7):100121. doi:10.1016/j.xcrm.2020.100121

52. Kim YI, Kim SG, Kim SM, et al. Infection and rapid transmission of SARS-CoV-2 in ferrets. *Cell Host Microbe*. 2020;27(5):704-709.e2. doi:10.1016/j.chom.2020.03.023

53. Palmer MV, Martins M, Falkenberg S, et al. Susceptibility of white-tailed deer (*Odocoileus virginianus*) to SARS-CoV-2. *J Virol*. 2021;95(11):e00083-21. doi:10.1128/JVI.00083-21

54. Zhao Y, Wang J, Kuang D, et al. Susceptibility of tree shrew to SARS-CoV-2 infection. *Sci Rep*. 2020;10(1):16007. doi:10.1038/s41598-020-72563-w

55. Hall JS, Knowles S, Nashold SW, et al. Experimental challenge of a North American bat species, big brown bat (*Eptesicus fuscus*), with SARS-CoV-2. *Transbound Emerg Dis*. 2020;00:1-10. doi:https://doi.org/10.1111/tbed.13949

56. Richard M, Kok A, de Meulder D, et al. SARS-CoV-2 is transmitted via contact and via the air between ferrets. *Nat Commun*. 2020;11(1):3496. doi:10.1038/s41467-020-17367-2

57. Fritz M, Rosolen B, Krafft E, et al. High prevalence of SARS-CoV-2 antibodies in pets from COVID-19+ households. *One Health*. 2020;11:100192. doi:10.1016/j.onehlt.2020.100192

58. Patterson EI, Elia G, Grassi A, et al. Evidence of exposure to SARS-CoV-2 in cats and dogs from households in Italy. *Nat Commun*. 2020;11(1):6231. doi:10.1038/s41467-020-20097-0

59. Ruiz‐Arrondo I, Portillo A, Palomar AM, et al. Detection of SARS‐CoV‐2 in pets living with COVID‐19 owners diagnosed during the COVID‐19 lockdown in Spain: a case of an asymptomatic cat with SARS‐CoV‐2 in Europe. *Transbound Emerg Dis*. 2021;68(2):973-976. doi:10.1111/tbed.13803

60. Temmam S, Barbarino A, Maso D, et al. Absence of SARS-CoV-2 infection in cats and dogs in close contact with a cluster of COVID-19 patients in a veterinary campus. *One Health*. 2020;10:100164. doi:10.1016/j.onehlt.2020.100164

61. Deng J, Jin Y, Liu Y, et al. Serological survey of SARS‐CoV‐2 for experimental, domestic, companion and wild animals excludes intermediate hosts of 35 different species of animals. *Transbound Emerg Dis*. 2020;67(4):1745-1749. doi:10.1111/tbed.13577

62. Xia H, Atoni E, Zhao L, et al. SARS-CoV-2 does not replicate in *Aedes* mosquito cells nor present in field-caught mosquitoes from Wuhan. *Virol Sin*. 2020;35(3):355-358. doi:10.1007/s12250-020-00251-0

63. Chen J, Huang C, Zhang Y, Zhang S, Jin M. Severe acute respiratory syndrome coronavirus 2-specific antibodies in pets in Wuhan, China. *J Infect*. 2020;81(3):e68-e69. doi:10.1016/j.jinf.2020.06.045

64. Michelitsch A, Hoffmann D, Wernike K, Beer M. Occurrence of antibodies against SARS-CoV-2 in the domestic cat population of Germany. *Vaccines (Basel)*. 2020;8(4):772. doi:10.3390/vaccines8040772

65. Fam BSO, Vargas-Pinilla P, Amorim CEG, Sortica VA, Bortolini MC. ACE2 diversity in placental mammals reveals the evolutionary strategy of SARS-CoV-2. *Genet Mol Biol*. 2020;43(2):e20200104. doi:10.1590/1678-4685-gmb-2020-0104

66. Wu C, Zheng M, Yang Y, et al. In silico analysis of intermediate hosts and susceptible animals of SARS-CoV-2. *ChemRxiv*. Published online April 3, 2020. doi:10.26434/chemrxiv.12057996.v1

67. Cao Y, Sun Y, Tian X, et al. Analysis of ACE2 gene-encoded proteins across mammalian species. *Front Vet Sci*. 2020;7:457. doi:10.3389/fvets.2020.00457

68. Liu Z, Xiao X, Wei X, et al. Composition and divergence of coronavirus spike proteins and host ACE2 receptors predict potential intermediate hosts of SARS-CoV-2. *J Med Virol*. 2020;92(6):595-601. doi:10.1002/jmv.25726

69. Bouricha EM, Hakmi M, Akachar J, Belyamani L, Ibrahimi A. *In silico* analysis of ACE2 orthologues to predict animal host range with high susceptibility to SARS-CoV-2. *3 Biotech*. 2020;10(11):483. doi:10.1007/s13205-020-02471-3

70. Buonocore M, Marino C, Grimaldi M, et al. New putative animal reservoirs of SARS-CoV-2 in Italian fauna: A bioinformatic approach. *Heliyon*. 2020;6(11):e05430. doi:10.1016/j.heliyon.2020.e05430

71. Gao S, Luan J, Cui H, Zhang L. ACE2 isoform diversity predicts the host susceptibility of SARS-CoV-2. *Transbound Emerg Dis*. 2021;68(3):1026-1032. doi:10.1111/tbed.13773

72. Li Y, Wang H, Tang X, et al. SARS-CoV-2 and three related coronaviruses utilize multipke ACE2 orthologs and are potently blocked by an improved ACE2-Ig. *J Virol*. 2020;94(22):e01283-20. doi:10.1128/JVI.01283-20

73. Wang Q, Qiu Y, Li JY, Liao CH, Zhou ZJ, Ge XY. Receptor utilization of angiotensin-converting enzyme 2 (ACE2) indicates a narrower host range of SARS-CoV-2 than that of SARS-CoV. *Transbound Emerg Dis*. 2021;68(3):1046-1053. doi:10.1111/tbed.13792

74. Halfmann PJ, Masato H, Shiho C, et al. Transmission of SARS-CoV-2 in domestic cats. *N Engl J Med*. 2020;383(6):592-594. doi:http://dx.doi.org/10.1056/NEJMc2013400

75. Farnia P, Aghajani J, Farnia P, et al. Evidence for SARS‑CoV‑2 circulating among stray dogs and cats: should we worry about our pets during the covid‑19 pandemic? *Biomed Biotechnol Res J*. 2020;4(5):S49-55.

76. World Organisation for Animal Health (OIE). COVID-19 Events in animals. OIE - World Organisation for Animal Health. Published April 30, 2021. Accessed August 25, 2021. https://www.oie.int/en/what-we-offer/emergency-and-resilience/covid-19/

77. Zhang Q, Zhang H, Gao J, et al. A serological survey of SARS-CoV-2 in cat in Wuhan. *Emerg Microbes Infect*. 2020;9(1):2013-2019. doi:10.1080/22221751.2020.1817796

78. Mathavarajah S, Dellaire G. Lions, tigers and kittens too: ACE2 and susceptibility to COVID-19. *Evol Med Public Health*. 2020;2020(1):109-113. doi:10.1093/emph/eoaa021

79. Shi J, Wen Z, Zhong G, et al. Susceptibility of ferrets, cats, dogs, and other domesticated animals to SARS–coronavirus 2. *Science*. 2020;368(6494):1016-1020. doi:10.1126/science.abb7015

80. Bosco-Lauth AM, Hartwig AE, Porter SM, et al. Experimental infection of domestic dogs and cats with sars-cov-2: pathogenesis, transmission, and response to reexposure in cats. *PNAS*. 2020;117(42):26382-26388. doi:10.1073/pnas.2013102117

81. Barr IG, Rynehart C, Whitney P, Druce J. SARS-CoV-2 does not replicate in embryonated hen’s eggs or in MDCK cell lines. *Euro Surveill*. 2020;25(25):2001122. doi:10.2807/1560-7917.ES.2020.25.25.2001122

82. Mou H, Quinlan BD, Peng H, et al. Mutations derived from horseshoe bat ACE2 orthologs enhance ACE2-Fc neutralization of SARS-CoV-2. *PLoS Pathog*. 2021;17(4):e1009501. doi:10.1371/journal.ppat.1009501

83. Zhou P, Yang XL, Wang XG, et al. A pneumonia outbreak associated with a new coronavirus of probable bat origin. *Nature*. 2020;579(7798):270-273. doi:10.1038/s41586-020-2012-7
