# Supplementary figures and images for: Investigating SARS-CoV-2 Susceptibility in Animal Species: A Scoping Review
Source: Environ Health Insights. 2022 Jun 28;16:11786302221107786. doi: 10.1177/11786302221107786 (PMC9247998; doi:10.1177/11786302221107786)

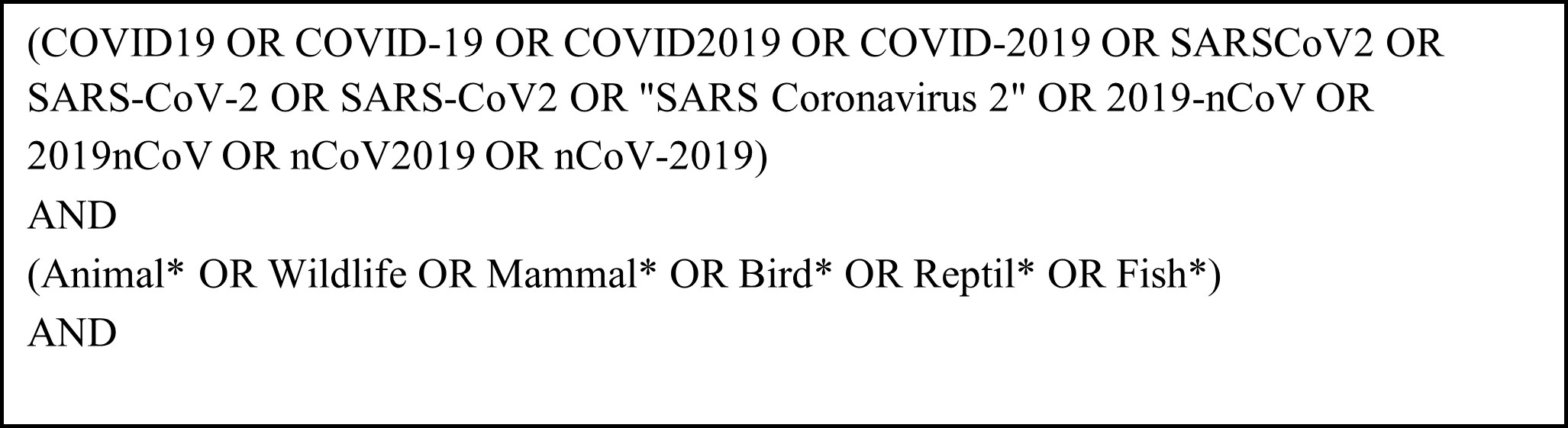

Supplement: sj-jpg-4-ehi-10.1177_11786302221107786 – Supplemental material for Investigating SARS-CoV-2 Susceptibility in Animal Species: A Scoping Review [file sj-jpg-4-ehi-10.1177_11786302221107786.jpg]
